# ASB7 Is a Novel Regulator of Cytoskeletal Organization During Oocyte Maturation

**DOI:** 10.3389/fcell.2020.595917

**Published:** 2020-11-05

**Authors:** Yuan Liu, Xiaoyan Li, Yongfu He, Hengjie Wang, Min Gao, Longsen Han, Danhong Qiu, Li Ling, Honglin Liu, Ling Gu

**Affiliations:** ^1^College of Animal Science and Technology, Nanjing Agricultural University, Nanjing, China; ^2^Jinling Hospital Department Reproductive Medical Center, Clinical School of Medical College, Nanjing University, Nanjing, China; ^3^State Key Laboratory of Reproductive Medicine, Nanjing Medical University, Nanjing, China

**Keywords:** oocyte, meiosis, ASBs, maternal aging, reproduction

## Abstract

Ankyrin repeat and SOCS box (ASB) family members have a *C*-terminal SOCS box and an *N*-terminal ankyrin-related sequence of variable repeats. To date, the roles of ASB family members remain largely unknown. In the present study, by employing knockdown analysis, we investigated the effects of ASB7 on mouse oocyte meiosis. We show that specific depletion of ASB7 disrupts maturational progression and meiotic apparatus. In particular, abnormal spindle, misaligned chromosomes, and loss of cortical actin cap are frequently observed in ASB7-abated oocytes. Consistent with this observation, incidence of aneuploidy is increased in these oocytes. Meanwhile, confocal scanning reveals that loss of ASB7 impairs kinetochore–microtubule interaction and provokes the spindle assembly checkpoint during oocyte meiosis. Furthermore, we find a significant reduction of ASB7 protein in oocytes from aged mice. Importantly, increasing ASB7 expression is capable of partially rescuing the maternal age-induced meiotic defects in oocytes. Together, our data identify ASB7 as a novel player in regulating cytoskeletal organization and discover the potential effects of ASB7 on quality control of aging oocytes.

## Introduction

Oocyte quality is crucial to female fertility. Mammalian primary oocytes are arrested at prophase of meiosis I, containing an intact nucleus, termed germinal vesicle (GV). Before ovulation, luteinizing hormone released by the pituitary gland stimulates the maturation of oocytes, leading to GV breakdown (GVBD), meiosis reinitiation, and chromosome condensation. At metaphase I, microtubules organize into a typical barrel-shape spindle, and all chromosomes line up on metaphase plate. The spindle migrates to peripheral cortical region of the oocyte, excluding first polar body (Pb1) after nuclear division and cytokinesis. Then, meiosis II occurs, and finally, oocyte is arrested at metaphase II until fertilization (Sen and Caiazza, 2013). The precise assembly of cytoskeleton and chromosomes during meiosis is essential to produce a normal matured oocyte. Errors at any step of this process may result in aneuploid eggs, which is the main cause of human spontaneous abortion, birth defects, and developmental disorders (Hassold and Hunt, 2001; Ma et al., 2014). The fertility of females decreases with age, and the frequency of aneuploid oocytes/embryos increases in old mammals (Golbus, 1981; Pan et al., 2008; He et al., 2019).

Ankyrin repeat and SOCS box (ASB) refers to the ankyrin repeat sequence and SOCS (suppressor of cytokine signaling) box family. ASB family has 18 members (ASB1-18), which are composed of two domains: a variable numbers of ankyrin repeats at its *N*-terminal and a SOCS box at the *C*-terminal (Kile et al., 2002; Mosavi et al., 2004). Ankyrin repeat is a 33-residue motifs in proteins, composed of two alpha helices separated by loops, and participates in multiple biological processes, such as cell cycle control (Lux et al., 1990), transcription factor activation (Pahl, 1999), cytoskeletal integrity, and ion transportation (Tee and Peppelenbosch, 2010). Most ASB proteins are a RING (really interesting new gene) family of E3 ubiquitin ligases (Anasa et al., 2018b). They can recruit target protein in the SOCS box domain for ubiquitination using ankyrin repeat sequence. ASB7 contains seven ankyrin repeats and is expressed in various tissues (Anasa et al., 2018b). Pharmacological induction of endoplasmic reticulum stress results in increased expression of ASB7 and simultaneously activates the unfolded protein response (Anasa et al., 2018a). Notably, a recent study has shown that ASB7 controls microtubule aggregation and chromosome stability in HeLa cells (Uematsu et al., 2016). To date, functions of ASB7 protein in meiotic oocytes have not been reported yet.

In the present study, we found that the depletion of ASB7 protein has an adverse effect on the meiosis of mouse oocytes, especially disrupting the assembly of meiotic apparatus.

## Results

### Subcellular Localization of ASB7 During Oocyte Maturation

**FIGURE 1:**
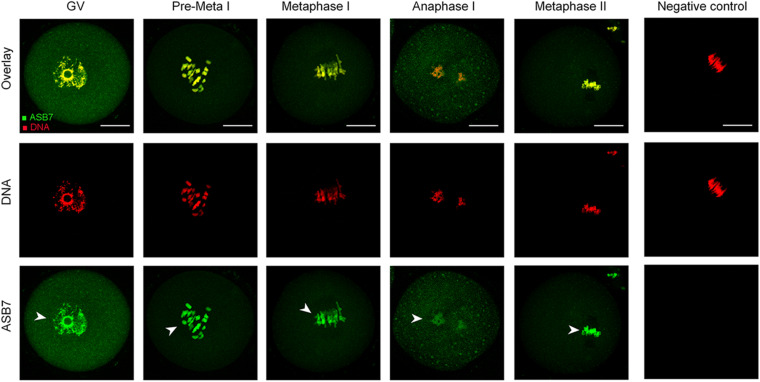
ASB7 subcellular localization in mouse oocyte. Oocytes at GV, Pre-Metaphase I, Metaphase I, Anaphase I, and Metaphase II stages were immunolabeled with ASB7 antibody (green) and counterstained with propidium iodide (PI; red) for DNA. Negative control without primary antibody was also included. Representative images were acquired under confocal microscopy. Arrowheads show the accumulation of ASB7 signal. Scale bar: 30 μm.

We first examined the localization of ASB7 protein in mouse oocytes at different stages by immunofluorescence staining. As shown in Figure 1, ASB7 is distributed in the entire GV oocytes and mainly accumulate in the nucleus (arrowhead). With the resumption of meiosis, ASB7 seems to be colocalized with the chromosomes from pre-metaphase I (Pre-MI) to metaphase II stage (arrowheads). Such a specific distribution pattern indicates that ASB7 may play an important role in the formation or stability of meiotic apparatus.

### ASB7 Knockdown Affects Meiotic Progression in Oocytes

**FIGURE 2:**
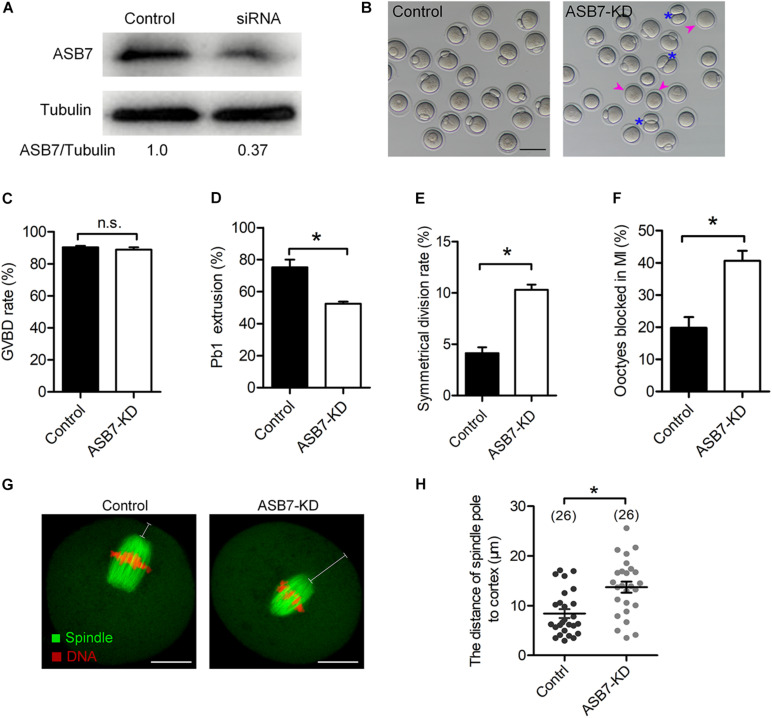
Effects of ASB7 knockdown on maturational progression of mouse oocytes. Fully grown oocytes microinjected with ASB7-siRNAs were arrested at GV stage with milrinone for 20 h. Negative control siRNAs were injected as control. **(A)** Western blots showing the efficient knockdown of ASB7 after siRNA injection, with tubulin as a loading control (100 oocytes per lane). **(B)** Phase-contrast images of control and ASB7-siRNA injected oocytes. Pink arrowheads indicate the oocytes that fail to extrude polar bodies and blue asterisks denote oocytes with apparent symmetric division. Scale bar: 100 μm. **(C–E)** Quantitative analysis of GVBD rate, Pb1 extrusion rate, and symmetrical division rate of control (*n* = 139) and ASB7-knockdown (*n* = 152) oocytes. **(F)** The percentage of oocytes arrested at metaphase I stage after ASB7-siRNA injection. Data are expressed as mean ± SEM from three independent experiments. **(G)** Control and ASB7-KD oocytes were sampled after 9.5-h culture and then stained with α-tubulin antibody to visualize spindle (green) and counterstained with PI to visualize chromosome (red). Scale bar: 30 μm. **(H)** The distance between the spindle pole and plasma membrane was quantified in the control and ASB7-KD oocytes. *p < 0.05 vs controls.

To explore the function of ASB7 in meiosis, the specifically designed ASB7 siRNAs were microinjected into fully grown GV oocytes. After injection, oocytes were arrested at GV stage for 20 h in the medium containing milrinone to allow the degradation of endogenous *Asb7* mRNA. Western blotting verified a significant knockdown of ASB7 protein in siRNA-injected oocytes (Figure 2A). The oocytes were transferred to normal medium for 3 h culture. Both control and ASB7 knockdown (ASB7-KD) oocytes resumed meiosis (Figure 2C). However, after 14 h culture, only 52.5% of the ASB7-KD oocytes extruded Pb1, which was lowered significantly compared with controls (Figures 2B,D, arrowheads). Meanwhile, we observed a high frequency of symmetrical division in ASB7-KD oocytes (Figures 2B,E, asterisks). In addition, by immunofluorescence staining and quantitative analysis, we found that 40.6% of ASB7-KD oocytes were arrested at MI, which was higher than controls (Figure 2F). The large polar body and symmetric division of oocytes is the main phenotype related to failure of spindle migration during meiosis I (Namgoong and Kim, 2016). In support of this notion, we confirmed that ASB7 knockdown hindered migration of spindle to cortical regions after 9.5 h culture (Figures 2G,H). Collectively, the results suggest that ASB7 knockdown disturbs the meiotic progression and cytokinesis during mouse oocyte maturation.

### Cytoskeletal Disorganization in Oocytes Depleted of ASB7

**FIGURE 3:**
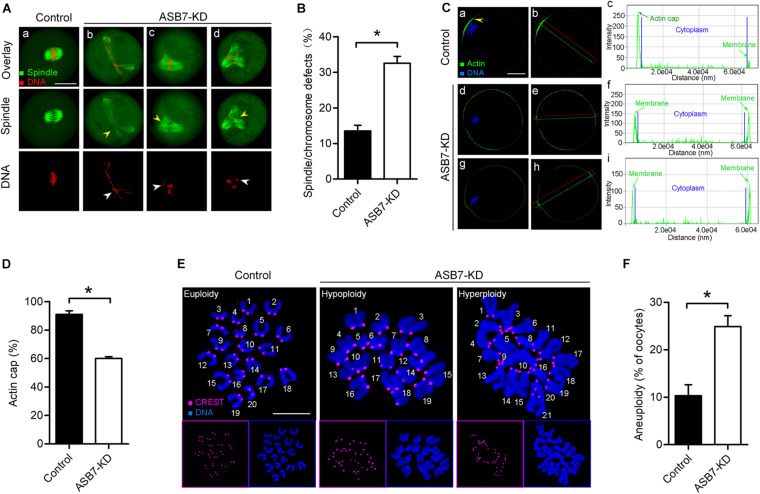
Depletion of ASB7 in oocytes disrupts cytoskeletal organization. **(A)** Control and ASB7-KD oocytes were stained with α-tubulin antibody to visualize spindle (green) and counterstained with PI to visualize chromosome (red). (*a*) Control oocytes show a representative barrel-shaped spindle and well-aligned chromosomes; (*b–d*) Disorganized spindles (yellow arrowheads) and misaligned chromosomes (white arrowheads) were readily observed in ASB7-KD oocytes. Scale bar: 30 μm. **(B)** Quantification of control (*n* = 140) and ASB7-KD (*n* = 152) with spindle/chromosome defects. **(C)** Metaphase oocytes were labeled with phalloidin to visualize actin (green) and counterstained with Hoechst 33342 for chromosomes (blue). Arrowhead shows the position of the actin cap in control oocytes. Profiles of actin fluorescence intensity along the green line in corresponding images are shown in the right panel (*c,f,i*). Scale bar: 30 μm. **(D)** Quantification of control (*n* = 32) and ASB7-KD (*n* = 35) with normal actin cap formation. **(E)** Chromosome spread of control and ASB7-KD MII oocytes. Chromosomes were stained with Hoechst 33342 (blue), and kinetochores were labeled with CREST (purple). Representative confocal images show euploid control oocytes and aneuploid ASB7-KD oocytes. Scale bar: 10 μm. **(F)** Quantification of aneuploidy in control (*n* = 43) and ASB7-KD (*n* = 50) oocytes. Data are expressed as mean percentage ± SEM of three independent experiments. *p < 0.05 vs controls.

The specific subcellular localization of ASB7 and its involvement in oocyte maturation promoted us to propose that ASB7 may play a role in the cytoskeleton disorganization. To test this possibility, control and ASB7-KD oocytes were immunolabeled with anti-tubulin antibody to check spindle and counterstained with propidium iodide to visualize chromosomes. Using confocal microscope, we noted that most control oocytes had a typical barrel-shaped spindle and well-aligned chromosomes on the metaphase plate. In contrast, the spindle defects and chromosome congression failure were readily observed in ASB7-KD oocytes (Figures 3A,B). Formation of cortical actin cap is crucial for polar body emission during oocyte meiosis (Sun and Schatten, 2006; Yi et al., 2013). To check whether ASB7 knockdown influences actin polymerization, ASB7-KD and control oocytes were loaded with actin tracker phalloidin and counterstained with Hoechst 33342. As shown in Figures 3C,D, actin caps were detected on the membrane of normal metaphase oocytes (arrowhead). Of note, profiles of fluorescence intensity clearly showed the failure to form actin cap in metaphase oocytes when ASB7 was abated. These abnormalities in meiotic apparatus in ASB7-KD oocytes might lead to the generation of aneuploid eggs. To test this hypothesis, we further analyzed the karyotype of MII oocytes by chromosome spreading and kinetochore immunolabeling. As shown in Figures 3E,F, we found an approximately twofold increase in aneuploidy incidence in ASB7-KD oocytes as compared to controls. The results indicate that the loss of ASB7 disrupts the cytoskeletal organization during oocyte maturation, which may be a major factor contributing to the meiotic defects we observed.

### Proper Attachments of Kinetochore–Microtubule Require ASB7

**FIGURE 4:**
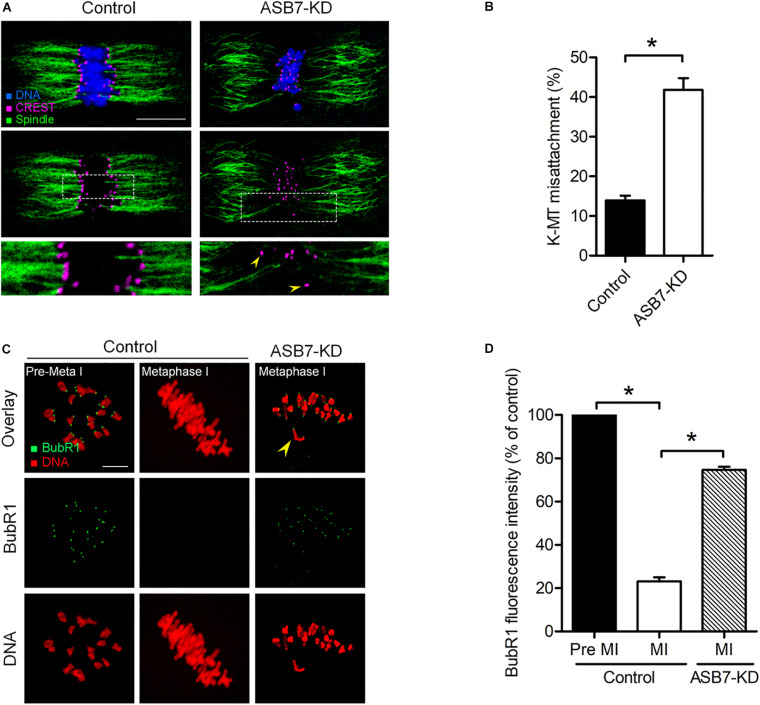
Loss of ASB7 in oocytes impairs kinetochore–microtubule (K–MT) attachments. **(A)** Control and ASB7-KD metaphase oocytes were labeled with CREST for kinetochores (purple), α-tubulin antibody for microtubules (green), and Hoechst 33342 for chromosome (blue). Representative confocal images are shown; arrowheads show misattached kinetochores. Scale bar: 10 μm. **(B)** Quantitative analysis of K–MT attachments in control (*n* = 48) and ASB7-KD (*n* = 55) oocytes. Kinetochores in regions where fibers were not easily visualized were not included in the analysis. **(C)** Control and ASB7-KD oocytes were immunolabeled with anti-BubR1 antibody (green) and counterstained with PI to examine chromosomes (red). Representative confocal images of pre-metaphase I and metaphase I oocytes are shown. Arrowhead indicates the scattered chromosome in ASB7-KD oocytes. Scale bar: 10 μm. **(D)** BubR1 fluorescence intensity in control (*n* = 46) and ASB7-KD (*n* = 42) oocytes was quantified. Data are expressed as mean percentage ± SEM of three independent experiments. *p < 0.05 vs controls.

Accurate chromosome movement involves the interaction between kinetochores and microtubules emanating from opposite spindle poles (Tauchman et al., 2015; Prosser and Pelletier, 2017). Considering the spindle/chromosome disorganization in ASB7-KD oocytes, we hypothesized that ASB7 may be required for correct kinetochore–microtubule (K–MT) attachments in meiotic oocytes. For this purpose, kinetochores were immunostained with CREST, spindle was labeled with anti-tubulin antibody, and chromosomes were visualized by Hoechst 33342. As shown in Figures 4A,B, the majority of control oocytes presented the typical amphitelic K–MT attachments. However, the proportion of K–MT misattachments in ASB7-KD oocytes was markedly increased compared with controls.

Cells have a sophisticated safety mechanism known as the spindle assembly checkpoint (SAC) to ensure that chromosomes have time to correctly line up on the spindle before the cell can divide (Lara-Gonzalez et al., 2012; Polanski, 2013; Marston and Wassmann, 2017). K–MT misattachments could be monitored and responded by the SAC. Therefore, we examined the SAC activity in oocyte by immunolabeling of BubR1 (budding uninhibited by benzimidazole-related 1), a pivotal part of the SAC system (Overlack et al., 2015). As shown in Figures 4C,D, in control oocytes, BubR1 was detected on unattached kinetochores at Pre-MI, but disappeared at MI when kinetochores were properly attached to microtubules. However, BubR1 signals on the kinetochores were remarkably elevated in those meiosis I-arrested ASB7-KD oocytes, indicating the activated SAC system. Cumulatively, these uncorrected K–MT attachments would lead to unstable biorientation of chromosomes and SAC activation, consequently contributing to the meiosis I block in ASB7-depleted oocytes.

### ASB7 Overexpression Alleviates Maternal Age-Induced Oocyte Meiotic Defects

**FIGURE 5:**
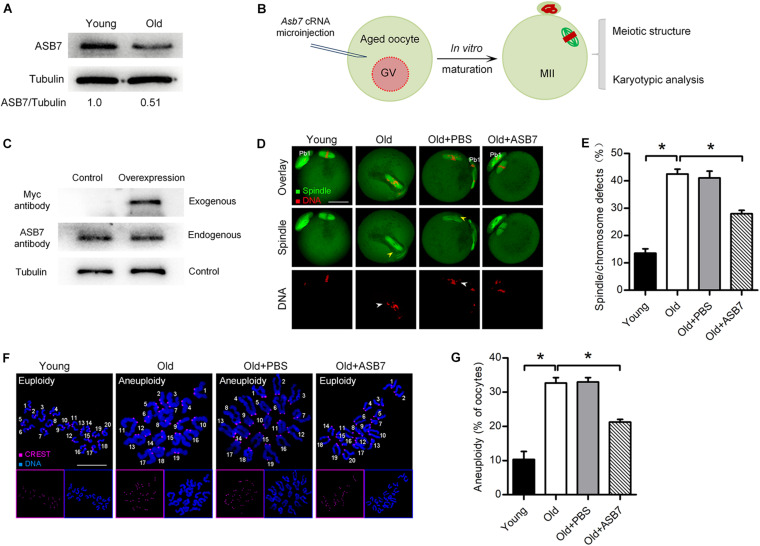
ASB7 overexpression alleviates maternal age-induced oocyte meiotic defects. **(A)** Representative Western blot showing the ASB7 expression in oocytes from young and old mice (100 oocytes per lane). **(B)** Schematic illustration of the experimental procedure for overexpression. **(C)** Representative Western blot result shows the efficiently overexpressed exogenous ASB7 protein. **(D)** Young, old, old + PBS, and old + ASB7 oocytes were stained with α-tubulin antibody to visualize spindle (green) and counterstained with PI to visualize chromosome (red). Yellow arrowheads direct to the disorganized spindles and white arrows indicate the misaligned chromosomes. Scale bar: 30 μm. **(E)** Quantification of young (*n* = 103), old (*n* = 112), old + PBS (*n* = 115), and old + ASB7 (*n* = 104) oocytes with spindle/chromosome defects. **(F)** Chromosome spread of young, old, old + PBS, and old + ASB7 MII oocytes. Chromosomes were stained with Hoechst 33342 (blue), and kinetochores were labeled with CREST (purple). Scale bar: 10 μm. **(G)** Quantification of aneuploidy in young (*n* = 35), old (*n* = 38), old + PBS (*n* = 34), and old + ASB7 (*n* = 32) oocytes. Data are expressed as mean percentage ± SEM of three independent experiments. *p < 0.05 vs controls.

It has been well recognized that female fertility decreases with maternal age due to the meiotic defects in oocytes (Liu and Keefe, 2002; Hunt and Hassold, 2008; He et al., 2019). Here, we found that ASB7-KD oocytes displayed similar phenotypes to those oocytes from aged mice. To check whether ASB7 is involved in the control of oocyte aging, we first evaluated its expression in oocytes from young (3–4 weeks) and old (42–45 weeks) mice. As shown in Figure 5A, a significant reduction of ASB7 protein was detected in oocytes from old mice compared with young oocytes. Next, we explored whether loss of ASB7 contributes to the defective phenotypes of aged oocytes by performing overexpression experiments (Figure 5B). cRNA encoding ASB7 was microinjected into fully grown GV oocytes from old mice. Western blotting confirmed that exogenous ASB7 protein was expressed in oocytes efficiently (Figure 5C). Consistent with previous reports (Nakagawa and FitzHarris, 2017; He et al., 2019), high frequency of spindle/chromosome anomalies were detected in aged oocytes. It is worth noting that these meiotic defects were partially rescued through the ectopic expression of ASB7 (Figures 5D,E). Moreover, in order to assess whether ASB7 overexpression in aged oocytes is also capable of preventing the generation of aneuploid eggs, karyotype analysis of MII oocytes was conducted by spreading chromosome. As shown in Figures 5F,G, correspondingly, the aneuploidy incidence in aged oocytes was significantly lowered when ASB7 expression was elevated. Altogether, these results suggest that loss of ASB7 is a critical factor contributing to the meiotic defects in aged oocytes.

## Discussion

Ankyrin repeat and SOCS box genes constitute a conserved chordate-unique gene family, which is defined as the substrate recognition subunits of Elongin–Cullin–SOCS (ECS) complexes (Kohroki et al., 2005; Okumura et al., 2012). In this study, we showed that ASB7 plays a novel role during mouse oocyte meiosis and discovered a beneficial effect of ASB7 overexpression on oocyte quality from aged mice. ASB7 protein is ubiquitously expressed in mammalian tissues and appears to be localized with Golgi, vesicles, and nucleus in cells (Anasa et al., 2018b). We found that the majority of ASB7 protein accumulated in the GV of immature oocytes. Accompanied by the resumption of meiosis, ASB7 protein appeared to be colocalized with chromosomes (Figure 1). Furthermore, we found that ASB7 depletion resulted in the failure of spindle assembly and chromosome congression in oocytes and aneuploidy generation (Figure 3), similar to the observations in HeLa cells (Uematsu et al., 2016). Kinetochore is a complex structure that establishes the attachment of spindle microtubules to chromosomes and is thus essential for faithful chromosome segregation (Santaguida and Musacchio, 2009). The correct chromosome alignment to metaphase plate requires bipolar attachment and tension between K–MT. Here, we noted that the K–MT interaction was disrupted in ASB7-KD oocytes (Figure 4). Kinetochore has been known as a signaling center that regulates chromosome attachment, SAC activity, and progression during metaphase-to-anaphase transition. Once there are unattached kinetochores, the SAC complex will be activated and inhibit anaphase onset (Tauchman et al., 2015). In line with this notion, the constant BubR1 signal on kinetochores and metaphase I arrest were observed when ASB7 was knocked down in oocytes (Figure 4). In addition, formation of actin cap was disturbed in ASB7-KD oocytes, indicative of the deficient actin polymerization (Figure 3). Altogether, our data support a model where ASB7, likely through the modulation of K–MT interaction, controls SAC signaling, consequently ensuring the proper assembly of meiotic apparatus in mammalian oocytes. Recently, it has been reported that the ASB7 protein regulates mitosis by targeting DDA3 (differential display and activated by p53) through proteasomal degradation (Uematsu et al., 2016). Future investigation is needed to clarify (i) whether this pathway is functional during mouse oocyte maturation and (ii) whether there are any other specific targets mediating the action of ASB7 on meiosis.

The decline of the female fertility is closely associated with age, largely due to the reduced developmental competence of eggs (Navot et al., 1994; Luke et al., 2012). For example, when women are over 35 years old, the risk of aneuploidy in oocytes is significantly increased, leading to infertility, abortion, and birth defects (Herbert et al., 2015). Although multiple pathways have been identified to be involved in maternal age-associated meiotic defects, the factors regulating meiotic structure remain to be discovered. In the present study, we observed that ASB7 protein levels are decreased in aged oocytes, showing the high frequency of spindle defects and chromosome misalignment. Of note, overexpression of ASB7 is capable of partially rescuing the defective phenotypes of old oocytes (Figure 5). Recently, a serial of proteins (i.e., SIRTs and HDAC3) have been reported to be responsible for the fertility loss of aged females (Ma et al., 2018; Yang et al., 2018; He et al., 2019; Nasheed Hamad Almohammed et al., 2020). Hence, dissection in greater depth of the possible interaction between ASB7 and these factors deserves further investigation.

In summary, this study reveals that ASB7 is a cytoskeletal regulator that is required for meiotic maturation in oocytes. Our work may provide clues for improving the *in vitro* maturation system and quality assessment of oocytes.

## Materials and Methods

All chemicals and culture media were purchased from Sigma (St. Louis, MO, United States) unless stated otherwise.

### Animals

Female ICR mice were used in all experiments, and 3- to 4-week mice were used as control. Mice that were 42–45 weeks old were selected as a reproductive aging model. All animal protocols were approved by the Animal Care and Use Committee of Nanjing Agricultural University, and all experiments were conducted in accordance with the guidelines of the local animal ethical committee and the Animal Care and Use Committee of Nanjing Agricultural University.

### Antibodies

Rabbit polyclonal anti-ASB7 antibodies were purchased from Novus Biologicals USA (Cat#: NBP1-86157; 1:250). Mouse monoclonal FITC-conjugated anti-α-tubulin antibodies were purchased from Sigma (Cat#: F2168; 1:500). Human anti-centromere CREST antibody was purchased from Antibodies Incorporated (Cat#: 15234; 1:500). Cy5 conjugated donkey anti-human IgG and FITC-conjugated donkey anti-goat IgG were purchased from Jackson ImmunoResearch Laboratory (Cat#: 709605149 and 705095147; 1:500). Goat polyclonal anti-BubR1 antibodies and mouse monoclonal anti-Myc-tag antibody were purchased from Abcam (Cat#: ab28193 and ab18185; 1:250). FITC-conjugated goat anti-rabbit IgG was purchased from Thermo Fisher Scientific (1:300).

### Collection and Culture of Oocytes

To retrieve fully grown GV oocytes, mice were superovulated with 5 IU Pregnant Mares Serum Gonadotropin (PMSG) by intraperitoneal injection, and 48 h later, cumulus-enclosed oocytes were obtained by manual rupturing of antral ovarian follicles. Cumulus cells were removed by repeatedly pipetting, and denuded oocytes were cultured in M16 medium under mineral oil at 37°C in a 5% CO_2_ incubator. For MII oocytes collection, mice were injected with 5 IU human Chorionic Gonadotropin (hCG) 2 days after PMSG priming. Oocytes were collected from oviduct ampullae 13.5 h post-hCG, and cumulus cells were removed by 1 mg/ml hyaluronidase incubation.

### Plasmid Construction and mRNA Synthesis

Total RNA was extracted from 50 denuded oocytes using the Arcturus PicoPure RNA Isolation Kit (Applied Biosystems, CA, United States), and cDNA was generated by Quantitect Purification Kit (Qiagen, Düsseldorf, Germany). PCR products were purified, digested with *Fse*I and *Asc*I (NEB Inc., MA, United States), and cloned into the pCS2^+^ vector with Myc-tags. For the synthesis of Myc-ASB7 mRNA, ASB7-pCS2 + plasmids were linearized by *Not*I, and capped cRNAs were *in vitro* transcribed with SP6 mMessage mMachine (Ambion, CA, United States) and purified by RNeasy Micro Kit (Qiagen, Germany). Synthesized RNA was aliquoted and stored at −80°C. The related primer sequences can be found in Supplementary Table 1.

### Knockdown and Overexpression Experiment

Microinjections of siRNA or mRNA were used to knock down or overexpress ASB7 in mouse oocytes, respectively. *Asb7* siRNA was diluted with water to a stock concentration of 1 mM, and then 2.5 pl siRNA solution was injected into oocytes, with negative siRNA injected as control. Ten picoliters of Myc-ASB7 mRNA solution (10 ng/μl) was injected into oocytes for overexpression experiment, with RNase-free PBS injected as controls. After injections, in order to promote mRNA to be degraded or translated, oocytes were arrested at GV stage in medium containing 2.5 μM milrinone for 18–20 h. Following three washes, oocytes were cultured in M16 medium without milrinone for maturation at 37°C in a humidified atmosphere of 5% CO_2_. The related *Asb7* siRNA sequences can be found in Supplementary Table 1.

### Immunofluorescence

Oocytes were fixed in 4% paraformaldehyde (PFA) for 30 min and then permeabilized with 0.5% Triton X-100 for 20 min. After three washes, oocytes were blocked in 1% BSA for 1 h at room temperature. Samples were incubated overnight at 4°C with primary antibodies and then at room temperature for 1 h with secondary antibodies. For F-actin staining, oocytes were fixed in 3.7% PFA for 5 min, blocked in 1% BSA for 1 h, and then incubated with FITC-conjugated phalloidin for 1 h. To detect kinetochores, oocytes were co-labeled with CREST. Chromosomes were counterstained with Hoechst 33342 (1:300) or propidium iodide (1:200) for 10 min. After three washes, oocytes were mounted on glass slides in a drop of anti-fade medium (Vectashield, Burlingame, CA, United States) and examined under a Laser Scanning Confocal Microscope (LSM 710 Carl Zeiss, Germany). Image J software (U.S. National Institutes of Health, Bethesda, MD, United States) was used to quantify the intensity of fluorescence.

### Western Blotting

A total of 100 oocytes were lysed in Laemmli sample buffer containing protease inhibitors and then heated for 5 min in boiling water. Samples were separated by 15% SDS-PAGE gel, transferred to PVDF membranes, and then blocked in PBST (PBS containing 0.1% Tween-20) with 5% low fat dry milk for 1 h at room temperature. The membranes were incubated with primary antibodies (Myc antibody, 1:1,000; ASB7 antibody, 1:400) at 4°C overnight. After washes in PBST, the membranes were incubated with HRP-conjugated secondary antibodies (1:2,000) for 1 h at room temperature. The protein bands were visualized using an ECL Plus Western blotting Detection System (GE Healthcare, Piscataway, NJ, United States). Membranes were reblotted with anti-α-tubulin antibody (1:5,000) for loading control.

### Chromosome Spread

Chromosome spreading was conducted as we previously described (Gao et al., 2018). MII oocytes were exposed to Tyrode’s buffer (pH 2.5) to remove zona pellucida, fixed in a drop of 1% PFA with 0.15% Triton X-100 on glass slide. Following air drying, samples were incubated with CREST (1:500) to label kinetochores at 4°C overnight, and chromosomes were stained with Hoechst 33342 (1:300).

### Statistical Analysis

Data are presented as mean ± SEM, unless otherwise indicated. Statistical comparisons were made with Student’s *t* test and ANOVA when appropriate. *p* < 0.05 was considered to be significant.

## Data Availability Statement

The raw data supporting the conclusions of this article will be made available by the authors, without undue reservation.

## Ethics Statement

The animal study was reviewed and approved by Animal Care and Use Committee of Nanjing Agricultural University.

## Author Contributions

YL and LG designed the research and analyzed data. YL, YH, HW, MG, DQ, LH, and LL performed the research. YH and LG wrote the manuscript. All authors contributed to the article and approved the submitted version.

## Conflict of Interest

The authors declare that the research was conducted in the absence of any commercial or financial relationships that could be construed as a potential conflict of interest.
