# Supplementary material for: ASB7 Is a Novel Regulator of Cytoskeletal Organization During Oocyte Maturation
Source: Front Cell Dev Biol. 2020 Nov 5;8:595917. doi: 10.3389/fcell.2020.595917 (PMC7674779; doi:10.3389/fcell.2020.595917)
Supplement: Supplementary file 1 [file Table_1.DOC]

# Supplemental Table 1

# Primer sequences of genes for cDNA amplification

*Gene Primer sequence*

*Asb7*

(1^st^ round) Forward primer: 5’-TGCTGACACTTCGGACCTGGTA-3’

Reverse primer: 5’-GGCTTGGACCTCATCGACTTGG-3’

*Asb7*

(2^nd^ round) Forward primer: 5’-GGGGGCCGGCCGATGTTACACCACCACTGCCG-3’

Reverse primer: 5’-GGGGGCGCGCCGTAATCTTGTCCACAGTCCAGGTG-3’

Primer sequences for siRNAs

*Gene Primer sequence*

*Asb7* Forward Primer: 5’-CCACGGUUAAGGAUUUAAUTT- 3’

Reverse Primer: 5’-AUUAAAUCCUUAACCGUGGTT-3’

Control Forward Primer: 5’-UUCUCCGAACGUGUCACGUTT-3’

Reverse Primer: 5’-ACGUGACACGUUCGGAGAATT-3’
